# Special Hospitals—Children

**Published:** 1892-10-01

**Authors:** 


### The Institutional Workshop.

### HOSPITAL ADMINISTRATION.

### III.—SPECIAL HOSPITALS—CHILDREN.

Cost of Management.*

Before we proceed to examine the figures in relation to the children's hospitals we must deal with a letter from Mr. Lennox Browne, which appeared in our issue of September 3rd, page 380. Mr. Browne is under a misapprehension in thinking that our note to Mr. Kershaw's letter (page 320) amounted to a charge of extravagance. What we want to arrive at is the minimum cost of treating an out-patient at a well-managed special hospital, say, for diseases of the throat. Mr. Browne argues that because the expenses of the out-patients at the fifty-five special hospitals printed with the statistics of the Hospital Sunday Fund averages 3s. 10¾d. per head, whereas the cost at the Central London Throat and Ear Hospital is only 3s. 6½d. per head, it cannot be maintained—as we understand him—that there is any justification for the statement that 3s. 6½d. is an excessive charge for an out-patient. We would point out, in the first instance, that the fifty-five special hospitals include every class. Thus the cost of an out-patient at the Cancer Hospital is given in these statistics as 27s. 8d., at the East-end Mothers' Home at 24s. 3d., and at St. Mark's Hospital for Fistula as 13s. 5d. for each out-patient. Several other special hospitals in the list return the cost of each out-patient at from 10s. to 8s. per head. To add up the cost of each out-patient at the fifty-five special hospitals, and to divide the total thus arrived at by the number of hospitals, irrespective of the class of work each institution does, and then to quote the average sum thus obtained as a fair index of what the expenditure at each of the many varieties of special hospitals should be, is obviously an untenable position to take up. This Mr. Lennox Browne will no doubt admit on reflection. Besides, the only fair way is to classify the various special hospitals into groups, and to compare the expenditure of those in each group, one with the other. This is the plan we have commenced, and we shall follow it to the end.

We must now pass on to the children's hospitals enumerated in the statistics of the Hospital Sunday Fund. There are twelve of these institutions altogether, if we include those which are practically homes for incurable patients. The number of beds they contain varies from 179 in the Hospital for Sick Children, Great Ormond Street, to 23 at the Belgrave Hospital. Another feature to be noticed is that the number of out-patients admitted to treatment seems to bear no relation to the number of beds, if we may judge from the fact that the Belgrave Hospital, with 23 beds, relieved only 2,141 out-patients, whereas the Paddington Green Hospital, with 27 beds, relieved no less than 11,509 out-patients in 1891. The authorities of the Belgrave Hospital return the cost per in-patient per week at £2 3s. 6d., and of each out-patient at 2s., whereas those at Paddington Green estimate the cost of each in-patient per week at £1 2s., and the cost of each out-patient at 1s. 9d. We propose to deal with the question of cost in a subsequent series of articles, and only mention the foregoing facts here in illustration of the difficulties which present themselves when an impartial attempt is made to judge the relative economy and efficiency of these institutions from the figures supplied by the hospital authorities.

We will now proceed to give a list of the Metropolitan Children's Hospitals.

Metropolitan Children's Hospitals.

| Name of Hospital. | Beds for Occupation. | Average Number Occupied. | Total In-Patients Admitted. | Total Out-Patients Admitted. | Percentage of Cost of Administration to that of Maintenance. |
|---|---|---|---|---|---|
| Hospital for Sick Children, Great Ormond Street | 179 | 130 | 1,495 | 20,112 | 13·509 |
| Victoria Hospital for Children | 120 | 68 | 1,081 | 15,241 | 16·877 |
| East London Hospital .. | 102 | 84 | 1,107 | 21,216 | 18·425 |
| Alexandra Hospital for Hip Disease | 81 | 81 | 154 | 90 | 10·715 |
| Evelina | 66 | 60 | 675 | 5,463 | 6·068 |
| North Eastern | 55 | 40 | 508 | 13,722 | 15·868 |
| Cheyne for Incurables .. | 50 | 50 | 72 | none. | 7·3 |
| Home for Sick Children, Sydenham | 45 | 44 | 221 | 1,551 | 3·0 |
| Home for Incurable Children, Maida Vale | 30 | 29 | 34 | none. | 6·755 |
| Paddington Green | 27 | 19 | 470 | 11,509 | 20·000 |
| Belgrave | 23 | 10 | 138 | 2,141 | 7·036 |
| The Vine, Sevenoaks ... | 17 | 16 | 28 | none. | 4·980 |

In considering the figures in the above table it is reasonable and fair to state that 15 per cent. in the case of general hospitals should be the maximum percentage of cost of administration to that of maintenance. There are no more popular institutions than hospitals for children, and none probably which elicit greater sympathy or a larger amount of spontaneous pecuniary support. Hence it cannot fairly be maintained that it is more difficult to raise money for the children's hospitals than for the general hospitals, and so 15 per cent. is undoubtedly the maximum expenditure upon management, which should be permitted by those who are responsible for the management of a hospital of this class. It is fair, however, to add that the situation of a children's hospital in the heart of a poor neighbourhood, far away from the public gaze, may render it necessary to expend somewhat more than 15 per cent., but in no case should the cost of management exceed 20 per cent. We are induced to make this qualification because it is essential that of all hospitals those for children should, as a rule, be situated in the heart of the poorest district.

A glance at the table shows that the largest sum spent upon management, that is, 20 per cent., by any children's hospital, is that of the Paddington Green institution. Situated where it is, and having regard to the comparative ease with which West-end charities raise their money, we think the authorities would do well to look into their expenditure with a view to seeing how they can reduce that upon management by at least 5 per cent. The East London Hospital for Children expends 18½ per cent. upon management, and the

* Previous articles of this series have appeared in The Hospital (page 365) for August 27th and (page 381) for September 3rd, 1892.

Victoria Hospital nearly 17 per cent. However legitimate and necessary the expenditure in the first case may be, we feel that the Victoria Hospital ought certainly to be able to reduce its expenditure upon management to 15 per cent. as a maximum. The cost of administration at the North-Eastern Hospital is nearly 16 per cent., but, like the East London, having regard to its situation in a poor district, we think it is highly creditable that they should be able so nearly to attain to the limit which experience indicates as desirable. There is one other feature which is somewhat remarkable. Whereas, in the case of the consumption hospitals, the cost of management doubled as the beds diminished, in the case of the children's hospitals it will be seen, generally speaking, that the fewer the number of beds the smaller is the percentage of cost of administration. Thus, only 3 per cent. is spent upon management at the Home for Sick Children, Sydenham, less than 5 per cent. at the Vine, Sevenoaks, and only 6 per cent. at the Evelina Hospital. These satisfactory results are probably due in the first two cases to the amount of voluntary assistance rendered to the authorities in raising money by persons interested in the work; and in the last to the fact that Baron Ferdinand de Rothschild practically finances the Evelina.

